# Carnivore Detection at the Domestic/Wildlife Interface within Mpumalanga Province, South Africa

**DOI:** 10.3390/ani11092535

**Published:** 2021-08-29

**Authors:** Jeanette Wentzel, Cory Gall, Mark Bourn, Juan De Beer, Ferreira du Plessis, Geoffrey T. Fosgate

**Affiliations:** 1 Production Animal Studies, Faculty of Veterinary Science, University of Pretoria, Onderstepoort 0110, South Africa; Vet@mtpa.co.za (F.d.P.); geoffrey.fosgate@up.ac.za (G.T.F.); 2 Wildlife Studies, Faculty of Veterinary Science, University of Pretoria, Onderstepoort 0110, South Africa; 3 Department of Veterinary Microbiology and Pathology, Washington State University, 2290NE, Westwood, #T204, Pullman, WA 99163, USA; gall.cory@gmail.com; 4 Mpumalanga Tourism and Parks Agency, Nelspruit 1200, South Africa; Mark.bourn@mtpa.co.za (M.B.); Juan.deBeer@mtpa.co.za (J.D.B.)

**Keywords:** camera traps, disease management, competition, epidemiology

## Abstract

**Simple Summary:**

The management of carnivore populations in protected areas includes disease management and census taking. The presence of prey species and environmental variables influence the detection of wild carnivore species. The aim of this study was to identify the important predictors of wild carnivore detection within two South African wildlife reserves using motion-detection camera traps. The study further investigated the difference between traditional census call-up surveys and camera traps within nearby locations. Buffalo, impala, and warthog were associated with lion and spotted hyena detections. Detections of lions and spotted hyenas and also leopards and spotted hyena were correlated, suggesting competition between these wild carnivore species. Competition among wild carnivore species has importance for implementing appropriate management procedures, including infectious disease prevention.

**Abstract:**

South African protected areas account for 8% of the total landmass according to World Bank indicators. Effective conservation of biodiversity in protected areas requires the development of specific reserve management objectives addressing species and disease management. The primary objective of the current study was to identify predictors of carnivore detection in an effort to inform carnivore species management plans on Andover and Manyeleti nature reserves in South Africa. A limited number of camera traps were placed randomly using a grid system. Species detection data were analysed using mixed-effects logistic regression and Spearman’s correlation coefficients. Deterministic inverse distance weighted distribution maps were used to describe the spatial distribution of carnivore species. Camera traps identified similar species as traditional call-up surveys during the study and would be useful as an adjunct census method. Carnivore detection was associated with several variables, including the presence of specific prey species. The measured intra-and interspecies interactions suggested the risk of disease transmission among species, and vaccination for prevalent diseases should be considered to manage this risk.

## 1. Introduction

South African protected areas, which include national parks and nature reserves managed by either national government, provincial government, or private landowners, total 8% of the country’s landmass (World Bank in August 2020). The conservation of biodiversity in protected areas should be guided by specific management objectives [1]. Biodiversity management must address both animals and plants [2], and relevant practices include veld (vegetation), water, species, and disease management. Biodiversity can drive competition and disease transmission among herbivore species [3,4,5], and the relative abundance of herbivore prey species influences the presence of carnivore species [6].

The 43% population decline of lions in 28 African countries (1968–2014) highlights the importance of carnivore management [7]. Carnivores are the apex species in the hierarchy and play an essential role in maintaining biodiversity within protected areas [8,9,10]. The social behaviour [11] of carnivores and the number of carnivores within the ecosystem [12] are important variables to consider to maintain the carnivore hierarchy. In addition to the ecological role of carnivores, their presence is also a valuable financial asset for protected areas in terms of photographic safaris and trophy hunting [13].

Africa has experienced a decline in carnivore populations, and wild lions are no longer present in seven African countries due to the lack of effective management [7]. Wild dog [14] and cheetah [15] populations have also declined, and both are currently listed on the IUCN Red List of Threatened species. The decline of carnivore numbers can be associated with human encroachment on protected areas [2]. Higher human populations hasten this encroachment and this in conjunction with poverty leads to human-wildlife conflict at the domestic/wildlife interface [16,17]. Potential conflict issues include crop damage and predation of livestock [18]. Human communities also raise domestic animals that share diseases with wildlife creating the possibility of disease spillover [19].

The presence of disease and changing environmental conditions can influence animal species diversity and abundance. Management plans should therefore include routine census taking as a priority. The reliable determination of species richness is essential to determine required management activities [20]. However, animal behaviour can limit the effectiveness of traditional animal census approaches since shy animals might not enter call-up sites [21]. Animal behaviour can therefore influence estimates of both richness and abundance within traditional carnivore surveys [22,23]. Camera traps can contribute to census data by collecting information to determine presence, absence, relative abundance, and also interactions among animals [24]. Camera trap data can improve the value of the traditional carnivore census [21], with 24 h recordings being cost-effective in terms of manpower [25]. Camera traps can also be used to monitor the domestic/wildlife interface to determine animal contact and disease transmission risk [22,26]. An added advantage of camera traps is that they can non-invasively monitor species diversity and animal behaviour [23,27]. Camera traps are especially effective for the detection of nocturnal and shy animals or animals in areas with low detectability [2]. Camera trap data have been used successfully to estimate relative abundance even in unmarked animals [28].

The aims of this study were to utilise camera traps to determine predictors of carnivore species detection, evaluate spatial overlap among species and descriptively compare results to traditional carnivore census methods on two protected areas within Mpumalanga Province, South Africa.

## 2. Materials and Methods

### 2.1. Study Location

The study area included two protected areas within Mpumalanga Province: one isolated (Andover Nature Reserve (NR)) and one part of the Greater Limpopo Transfrontier Conservation Area (GLTFCA; Manyeleti NR). These nature reserves are 51 km apart (main camp to main camp) and are both managed by Mpumalanga Tourism and Parks Agency (MTPA). The two reserves are in the Lowveld, Manyeleti, on the western boundary with the Kruger National Park (KNP), with Orpen as the nearest rest camp. Andover is 51 km away towards Kamperus and not part of the Greater Kruger National Park. Protected area (A), Andover NR was 7000 ha in size, with the main camp located at: S: −24.582128, E: 31.228589. Andover NR was bordered by human settlements on the southeast, south, and western boundaries and was situated in the Granite Lowveld vegetation type, which is characterized by undulating landscape with interspersed drainage lines [29]. The upper landscapes aligned in a westerly to an easterly direction and received annual precipitation of 783 mm [30]. Carnivores present on Andover NR included leopard (*Panthera pardus*) and spotted hyena (*Crocuta crocuta*) with the occasional lion (*Panthera leo*) entering from adjacent properties. General game species present included buffalo, giraffe, zebra, waterbuck, and kudu [31].

Protected area (B), Manyeleti NR, was 22,600 ha in size, with the main camp located at: S: −24.580309, E: 31.339229. Manyeleti NR shared open boundaries with private nature reserves in the northwest and south whilst also sharing an open boundary with the KNP and the GLTFCA on the north and east. Manyeleti NR was situated in the Savanna region of the Lowveld and had two different veld types, namely Gabbro Grassy Bushveld and Granite Lowveld [29]. Manyeleti NR mainly consisted of woody vegetation, including grasses, shrubs, and trees with an annual summer rainfall varying from 500 mm in the north to 700 mm in the south [32]. The average daily maximum temperatures varied between 30 °C in the summer months (Jan) and 23 °C in winter (July), with an altitude from 347 to 499 m above sea level. There were 17 human settlements situated between Andover and Manyeleti NR that encompassed 29,500 ha with approximately 40,000 people in 8500 households [33].

Manyeleti NR western border acted as the domestic/wildlife interface and was designed to comply with the veterinary procedural notice concerning buffalo disease control and the Biodiversity Management plan in terms of the National Environmental Management: Biodiversity Act (NEMBA), Act 10 of 2004 [34,35].

### 2.2. Field Methodology

The two study sites were divided into grid cells using Google Earth maps (https://earth.google.com/web/ accessed on 2 January 2015), with Andover NR divided into a total of 83 grid cells (1 × 1 km^2^ each) while Manyeleti NR was larger and subsequently divided into 82 grid cells (2 × 2 km^2^ each). The number of grid cells did not correspond to the reported areas due to the irregular shape of the reserves and the requirement to cover the entire area. Short-term camera traps were placed within randomly selected grid cells, and long-term camera traps were placed at the domestic/wildlife interface on the wildlife side of the fence. Three to four camera traps (Cuddeback Attack IR, Model 1158, Non Typical, Inc., Green Bay, WI, USA) were placed within selected grid cells to ensure coverage of all vegetation types present within the grid cell.

**Figure 1:**
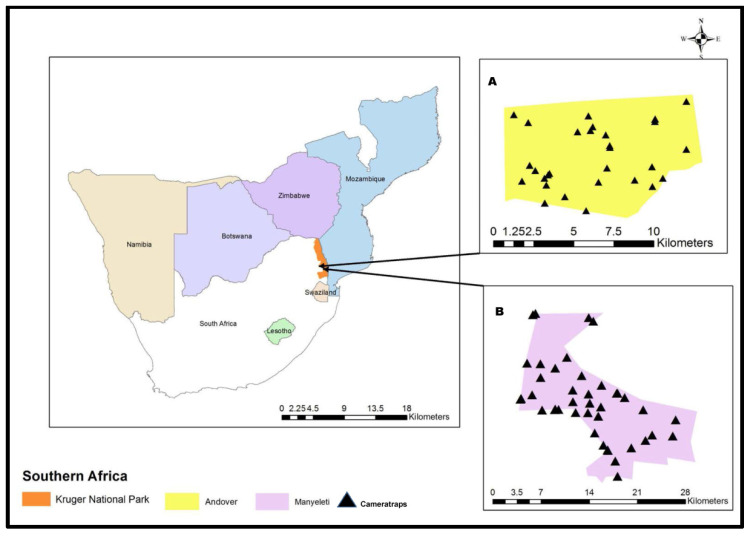
Camera trap locations in Andover (**A**), and Manyeleti (**B**) nature reserves, Mpumalanga Province, South Africa from 2015–2017.

Each camera trap was secured to a natural structure (e.g., tree) 1.2–1.5 m above ground level and angled downwards. Camera traps were set up to be away from direct morning sunlight and enclosed within an outer metal box to reduce animal contact and subsequent damage. Camera traps were deployed using a motion trigger setting. Each camera trap location was Geo-referenced using a handheld GPS (Garmin Etrex 10, Garmin Ltd., Onderstepoort, South Africa) and a panoramic photograph was taken to describe the vegetation type and topography at the time of camera trap deployment. Researchers visited camera trap locations once a week to inspect for physical damage and download data. Camera traps were deployed from February 2015 until June 2017 (Figure 1).

### 2.3. Data Collection

The predominant vegetation at camera trap locations was described using Edwards’ criteria that included woody cover, scrubs, and grasslands [36]. Weather information was collected from stations within the protected areas and verified using data from the closest South African Weather Station. Collected meteorological data included daily minimum and maximum temperatures (°C) and rainfall (daily in mm). Summer was defined as December to February, autumn consisted of March to May, winter comprised of June to August, and spring was September to November. Data concerning distances to fence lines and roads, along with the moon phases, were also collected. The study focused on wild carnivore species, including lion (*Panthera leo*), African leopard (*Panthera pardus*), cheetah (*Acinonyx jubatus*), African wild dog (*Lycaon pictus*), serval (*Leptailurus serval*), slender mongoose (*Galerella sanguinea*), spotted hyena (*Crocuta crocuta*), African civet (*Civettictis civetta*), small spotted genet (*Genetta genetta*), side-striped jackal (*Canis adustus*), and caracal (*Caracal caracal*). Additional data concerning prey and domestic animal (dogs, cows, donkeys) detection inside and outside the reserves were also collected.

### 2.4. Data Summarization

Raw data were summarised to determine species detection on a specific day at a specific camera trap site, and the rate of detection (catch per unit effort) was calculated as the total number of detections divided by the total number of days each camera trap was functioning at a certain location. Detection rates per day for individual carnivore species and carnivore groups were calculated. The feline carnivore group included lion, leopard, cheetah, serval, and caracal; the canine carnivore group included African wild dog and jackal; and the other carnivore group included spotted hyena, genet, and civet.

### 2.5. Census

Biannual call-up surveys were performed during the study period (2015–2017). Call-up surveys were done following established guidelines [21], and species diversity was recorded per site. Camera trap detections on the day of the call-up survey and within a 5 km radius were used for descriptive comparisons. Since camera trap locations were random, the number of camera traps within this 5 km range varied between 1 and 4 on census days. Reserve management was responsible for general game and carnivore censuses. When budget allowed, aerial game censuses were performed, and when not possible, game numbers were estimated by using population growth modelling.

### 2.6. Data Analysis

#### 2.6.1. Univariate Predictors of Carnivore Species Detection

All statistical analyses were performed using SPSS Version 25.0 (International Business Machines Corp., Armonk, NY, USA), and significance was set as *p* < 0.2 during initial variable screening as is commonly employed for epidemiological model building. Daily carnivore detection (detected/not detected) data were analysed using mixed-effects binary logistic regression. Models included a random effect term for camera trap location and predictors screened for an association with carnivore species detection. The odds ratio (OR) was used to estimate the influence of the variable on the detection of a specific carnivore species or group. The 95% confidence interval (CI) was used to measure the precision of the OR. A wide CI indicated low precision, and a narrow CI indicated high precision [37].

#### 2.6.2. Multivariable Predictors of Carnivore Species Detection

Multivariable mixed-effects logistic regression models were fit to estimate adjusted measures of association between studied predictors and detection of carnivore species and groups. All predictors that were *p* < 0.2 within the univariate screening models were entered into starting multivariable models. When collinearity was present among predictor variables (for example, total rain over the past 1 day, 2 days, etc.), then only the single variable with the strongest screening association was chosen for the initial multivariable model. The camera trap location was included as a random effect, and a fixed effect for each reserve was forced into models to adjust for potential confounding. Variables were subsequently removed in a manual stepwise process based on the largest coefficient *p*-value. The stepwise process continued until the significance level of all remaining coefficients was *p* ≤ 0.05. Independent models were fit for the detection of all study carnivore species and groups. Statistical modelling was performed using commercially available software (SPSS Version 25.0, International Business Machines Corp., Armonk, NY, USA).

### 2.7. Interspecies Correlation

Summarized raw daily counts for all camera trap locations were analysed. The rate of species detection was calculated as the total number of observations for each species or carnivore group divided by the total number of camera trap days. Detection rates were assessed for normality by calculating descriptive statistics, plotting histograms, and performing the Anderson–Darling normality test in commercially available software (MINITAB Statistical Software, Release 13.32, Minitab Inc., State College, PA, USA). Spearman’s correlation coefficient (rho) was used to estimate the interspecies correlation for the rates of detection at each camera trap location among carnivore species and between prey species due to the apparent violation of the normality assumption. Correlations were determined using commercial software (SPSS Version 25.0, International Business Machines Corp., Armonk, NY, USA), and significance was set as *p* ≤ 0.05. Estimated correlations were interpreted using four classifications: Strong (≥0.8), moderate (0.6 to 0.7), fair (0.3 to 0.5), and poor (<0.3) [38].

### 2.8. Spatial Interpolation

ArcGIS version 10.4.1 (ESRI, Redlands, CA, USA) was used to plot camera trap locations on the study reserves in relationship to KNP and human settlements. Maps were created using a scale of 1: 250,000 and projected using GCS WGS 1984 as the coordinate system. Species distribution maps were created using the summarized rate of detection (counts/day) and performing deterministic inverse distance weighting (IDW).

## 3. Results

### 3.1. Camera Traps

A total of 12 camera traps were deployed during the study covering 40 locations in Andover NR and 51 locations in Manyeleti NR. Andover NR had one long-term camera trap while Manyeleti NR had three long-term camera traps. The average number of camera trap days for long-term locations was 487 with an average of 46 days for the randomly selected short-term sites. A total of 6435 camera trap days of data were collected over the whole study. On average, 103 pictures were taken per day with an overall total of 74,829 photos. Carnivores were detected within 440 photos while there were 8619 photos of noncarnivore animal species. The 440 carnivore photos were comprised by the following species (numbers for Andover NR, numbers for Manyeleti NR): spotted hyena (n_A_ = 27; n_B_ = 195), lion (n_A_ = 0; n_B_ = 61), leopard (n_A_ = 16; n_B_ = 41), wild dog (n_A_ = 0; n_B_ = 33), jackal (n_A_ = 1; n_B_ = 8), serval (n_A_ = 8; n_B_ = 15), civet (n_A_ = 2; n_B_ = 9), caracal (n_A_ = 4; n_B_ = 6), mongoose (n_A_ = 5; n_B_ = 6), and cheetah (n_A_ = 0; n_B_ = 3).

### 3.2. Census and Comparision with Camera Traps

During the study period, five biannual call-up surveys were performed that identified 56 lions, 14 leopards, 44 spotted hyenas, three wild dogs, two cheetahs, and one jackal. In comparison, the overlapping camera traps (within a 5 km radius with the applicable call-up surveys at the same time) identified 65 lions, 12 leopards, 75 spotted hyenas, 5 wild dogs, one cheetah, and three jackals. Some call-up days were similar to the camera trap detections, while others differed more substantially with higher numbers in either the survey or the camera trap detections. One example was at night Call Station 6, October 2016 (Supplemental data: Table S1), where the camera traps detected nine lions and nine spotted hyenas. However, only two lions and one leopard were called in during the survey. At Call Station 5 (October 2015), six lions and five spotted hyenas were identified during the call-up survey, while the camera traps only detected one lion and three spotted hyenas. Call Station 1 (October 2016), the camera traps detected 28 lions, two leopards, 12 spotted hyenas, one jackal, and five wild dogs, while only three lions were identified by the call-up survey on the same night.

### 3.3. Variables Predicting Carnivore Detection

Several variables were significant predictors of carnivore detections based on univariate screening models (Supplemental Tables S2–S6). Multivariable models identified summer and the presence of vehicles as significant predictors for the detection of any carnivore (Table 1).

The three most important prey species that increased the detection of any carnivore species were impala, followed by buffalo and warthog. Feline carnivores were more likely to be detected when hippos and hares were also detected at the same location. No evaluated variables were significant predictors of canine carnivore detection during the study. The availability of certain prey species influenced the detection of other carnivore species.

### 3.4. Interspecies Correlation

The detection of lions at specific sites was positively correlated with the detection of spotted hyena and cheetah, but the detection of leopards and lions were not significantly associated with each other (Table 2).

Leopard detection was positively correlated with spotted hyenas and jackals. Jackal detection was also positively correlated with the detection of spotted hyenas. The detection of serval was positively correlated with the corresponding detection of caracal and civet. The most significant carnivore-prey correlations were lions with buffalo, wildebeest, impala, and warthog. The detection of leopards was correlated with the detection of giraffe and waterbuck, while spotted hyena was positively correlated with giraffe, rhino, buffalo, wildebeest, impala, and warthog.

### 3.5. Preferred Locations of Carnivores

**Figure 2:**
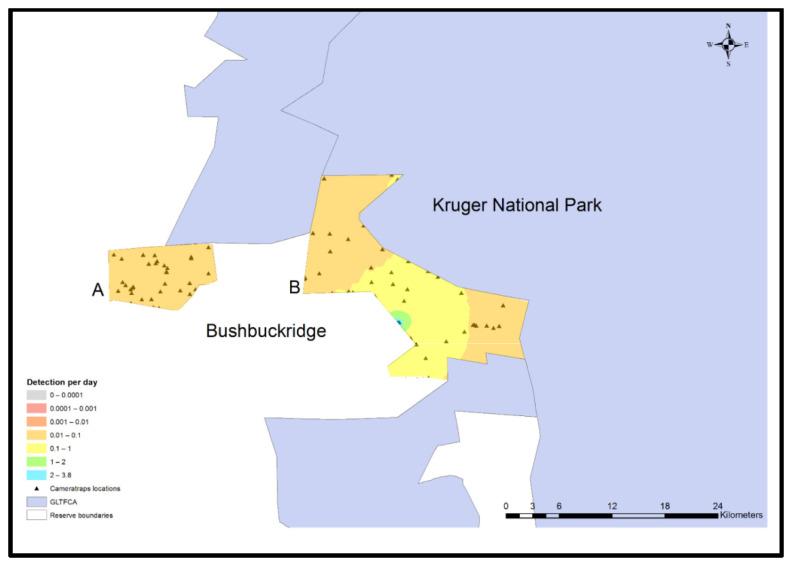
Any carnivore (lion, leopard, serval, civet, cheetah, jackal, wild dog, genet, mongoose, and spotted hyena) daily detection model for Andover (**A**) and Manyeleti (**B**) nature reserves adjacent to Bushbuckridge human settlements and Kruger National Park, Mpumalanga Province, South Africa, 2015–2017.

**Figure 3:**
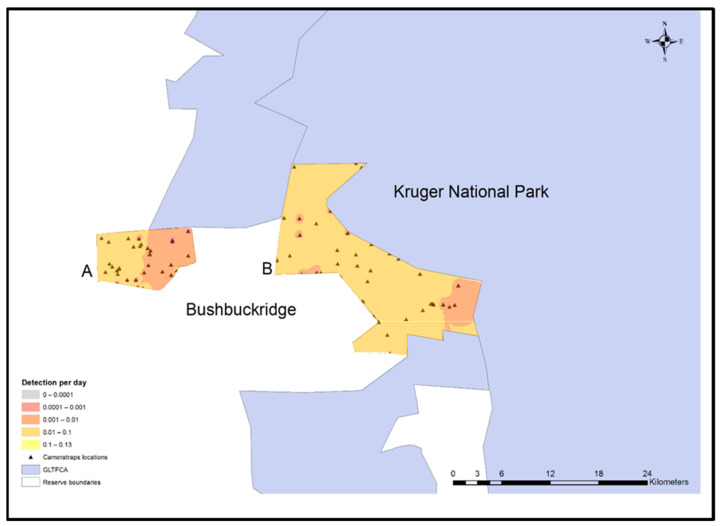
Any feline carnivore (lion, leopard, cheetah, and serval) daily detection model for Andover (**A**) and Manyeleti (**B**) nature reserves adjacent to Bushbuckridge human settlements and Kruger National Park, Mpumalanga Province, South Africa, 2015–2017.

The rate of all carnivore detection in Andover NR was negligible, whereas the central portion of Manyeleti NR was the most probable area for detecting carnivores (Figure 2). Feline carnivore species were rarely detected in either reserve (Figure 3).

**Figure 4:**
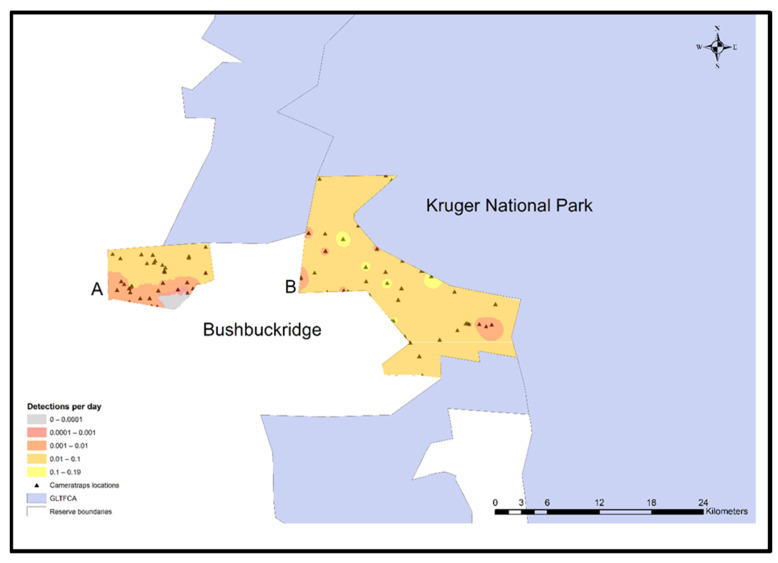
Other carnivore (spotted hyena, civet, and genet) daily detection model for Andover (**A**) and Manyeleti (**B**) nature reserves adjacent to Bushbuckridge human settlement and Kruger National Park, Mpumalanga Province, South Africa, 2015–2017.

Although the detection rate of other carnivores (mainly spotted hyena) was low in general, this group seemed to prefer the northern fences with the neighbouring nature reserves and away from the communal areas associated with Andover NR (Figure 4).

## 4. Discussion

The two protected areas were both managed by MTPA using the same regional ecologist. However, each reserve had its own onsite management team (reserve manager, conservation manager, and rangers). The tourism industry in sub-Saharan Africa has been estimated to be worth US$25 billion [39] and plays an important role in conservation. Species diversity encourages photographic tourism, and tourism tends to be in areas with higher species densities [40]. Carnivore presence can be utilised as a selling point for tourism [41].

On Andover NR, only a few camera trap sightings of solitary leopard and spotted hyena were recorded, with the majority of carnivore observations being the smaller species such as serval, genet, and civet. This suggests low apex predator numbers on this reserve and with a majority being meso-predators. This could have a negative impact on the biodiversity hierarchy of Andover NR. In a similar study on the southern California coastline, a decline in coyotes (*Canis latrans*) allowed for an increase in gray foxes (*Urocyon cinereoargenteus*) and domestic cats, and this disrupted hierarchy caused a large reduction in shrub breeding bird populations [42].

Different methods are available to estimate carnivore density, including direct counts, camera traps, distance sampling, and genetic surveys [43]. The most general and cheapest in terms of technology are track surveys and call-up surveys [44]. Call-up surveys are reliable to monitor carnivore populations in the long term if approximately 20% of the protected area can be covered. However, estimates must consider that animals on a kill and feeding females with cubs will not be drawn into the call-up site [21]. Although call-up surveys have been used for years, alternatives are available to either support [21] or replace traditional call-up surveys. Capture-recapture methods using camera traps can be an improvement over the traditional call-up survey [22,45]. Camera traps can be used to validate call-up survey sites and add additional information to the call-up survey technique. Camera traps can identify nocturnal and diurnal carnivore presence in multiple locations and potentially reduce the inherent bias with call-up surveys [46]. During this study, the five census call-up surveys between 2015–2017 were expected to call in predators within a 5 km radius as per guidelines [21]. The results obtained from deployed camera traps and the call-up surveys were often different, and predictors of carnivore detection might therefore be useful to determine more effective call-up survey locations.

Carrying capacities and carnivore presence are influenced by various predictors, including environmental conditions [2,47]. Such predictors could be used to manage carnivore populations for species monitoring and moving carnivore species into new locations using non-invasive techniques. For example, our results reinforced the notion that carnivore species are prey-driven, with carnivore presence associated with the detection of impala, buffalo, and warthog in the same locations. Also, there was an increase in carnivore species detection during the spring, and this is probably because it coincides with impala lambing season, buffalo calving season, and most carnivore births. The finding that carnivore species detection correlates with specific prey species is consistent with a KNP study of five carnivore species (lion, spotted hyena, leopard, cheetah, wild dog) at 22 prey kill sites. Both lion and spotted hyena were identified as hunting megaherbivores that included hippo and elephant. However, impala were the main prey species for all carnivores comprising 14% of lions, 40% of spotted hyenas, and up to 70% of leopard, cheetah, and wild dog kills [6].

In this study, the feline carnivore group (mostly lion) detections were significantly associated with the presence of buffalo and hippo. This finding is similar to previous research conducted in KNP and other South African reserves [6,48,49,50,51]. Also, the unusually dry season during the study (El Nino 2015–2017) [52,53] might have caused prey specialisation to occur as a result of prey congregation [54]. The presence of hares was also associated with the detection of the feline carnivore group (lion, leopard, cheetah, and serval), and this is consistent with a kill site study in the Kalahari that reported leopard hunting scrub hare [55]. Additionally, behavioural studies have reported that leopard, cheetah, and serval use hares to train juveniles how to hunt [11]. A previous study reported that lions and cheetah also preyed on kudu [6], but this association was not identified in the current study. This was possibly due to the relative abundance of impala and buffalo with few kudus within the study locations. A previous review reported that spotted hyenas tend to avoid areas with buffalo, zebra, and giraffe [56]. This is in contrast to the results of the present study that identified a positive association between buffalo presence and the detection of spotted hyenas. The results of the current study are also similar to an earlier study in KNP that reported that the diet of spotted hyenas included buffalo, warthogs, and impalas [57].

The study locations with the highest detection of carnivore species were in the middle of the Manyeleti NR, and this could be due to easy accessibility to water resources (Maindam area) [58]. However, this also had the shortest distance to the domestic/wildlife interface and domestic prey [59]. The influence of prey should not be underestimated because the movement of prey can influence the presence of carnivores, in addition to the potential disease risk associated with prey selection. Patch burns were employed as veld management [60] within Manyeleti NR during the study, and the forced movement of herbivores might have indirectly influenced the presence of carnivores in certain locations.

No evaluated landscape variables were significant predictors of carnivore detection except for elevation predicting spotted hyena detections. However, elevation within the study area only varied between 378 and 571 m above sea level. Spotted hyenas were reported to prefer areas around 300 m within the Majete Wildlife Reserve (Malawi), and this was attributed to prey availability and vegetation transition of these areas [61]. Prey availability was controlled for within the multivariable models of the present study, and further investigation is therefore required to investigate the reasons for this association.

The presence of safari vehicles increased the detection of carnivore species, which might be due to a combination of factors. One factor might be that daily game drives in the study area caused carnivore species, such as spotted hyenas, to become habituated to vehicles. Additionally, experienced guides might drive to areas with a higher likelihood of seeing carnivore species. This finding contradicts a previous behavioural study in the Masai Mara Nature Reserve (Kenya), which reported that areas with higher vehicle tourism were avoided by carnivore species, including spotted hyena [62].

Interspecies competition can be used as an indicator of carnivore species diversity [3,63,64,65]. The significant correlation between lion, leopard, spotted hyena, cheetah, wild dog, and other carnivores indicated shared habitat use; however, the correlation varied between weak and fair among the different species combinations. Competition and spatial overlap can lead to possible disease transmission either by direct or indirect contact [66].

Blood parasites are transmitted via vectors including *Amblyomma hebreum*, *Rhipicephalus sanguineus,* and *Heamophysalis elliptica* ticks that have life stages that can survive in the environment [67]. Multi-host ticks might be shared among carnivore species creating the possibility for disease transmission [67]. Tick control could be used to reduce this disease transmission risk, and in a closed system such as Andover NR, it is possible by applying parasiticides or tick deterrents [68]. For example, a pour-on parasiticide can be applied using a saturated treatment column while the animals eat an attractant lick [68]. Another possibility is an artificial spray race along an animal path that is activated when animals step upon a touch plate. However, “no interference policies” and limited budgets will influence the use of these management tools. Controlled burns can also be used, but fire only has short-term effects as ticks re-establish after animals return to these areas.

Disease impacts must be managed to ensure the survival and conservation of endangered species with limited gene pools, such as leopard, cheetah, and wild dog [15,69]. Rabies in endangered species is an important consideration that can be prevented through the use of oral vaccinations or dropout darts [70,71,72]. Reserves that border domestic areas can also encourage the vaccination of domestic animals for rabies in the area, and this approach was effective in the Serengeti [73,74]. Additionally, interactions between carnivore and herbivore species can contribute to disease transmission in protected areas. Buffalo is an intermediate host of Toxoplasma [75] and a carrier of bovine tuberculosis [76]. Impala, warthog, waterbuck, and wildebeest can also be affected by bovine tuberculosis [77], Neospora [78] and serve as intermediate hosts of Toxoplasma [79].

Wild felids are considered definitive hosts of *Toxoplasma gondii,* with a lion in the Serengeti being the first reference to an infected wild carnivore [79]. Many wild herbivores can become infected with *Toxoplasma gondii* and present exposure risks to carnivores [80]. *Toxoplasma gondii* infection can cause abortions in ungulates that might expose scavenging carnivores. There is limited information available on the impact of *Toxoplasma gondii* in wild ecosystems, but based on data from domestic species, it can be assumed that oocyst shedding is restricted to a limited period following primary infection. Therefore, smaller carnivores with shorter lifespans might be more important epidemiologically. Small felids including African wildcat, serval, and caracal are therefore expected to have a higher impact on environmental contamination with *Toxoplasma gondii* oocysts compared to lions, leopards, and cheetahs [3,81,82]

Effective fencing should prevent contact between domestic carnivores and wild carnivores and thus mitigate disease transmission risk at the domestic/wildlife interface. Rabies and canine distemper can be transmitted by direct contact with an infected domestic dog at the domestic/wildlife interface [83,84], and an effective fence should reduce this probability of contact. Toxoplasma can also be transmitted via the consumption of infected domestic prey, including cattle [85], and these interactions will also be reduced by effective fencing. The interaction of wild carnivores with domestic dogs at the interface is a threat to the survival of wild carnivores [4], and reduced contacts would be expected to reduce disease transmission risk. The disease risk at the domestic/wildlife interface is unpredictable but can influence carnivore populations if not well managed [86,87]. In this study, no direct contact was observed between domestic and wild carnivores despite both being detected at the fence line of the domestic/wildlife interface.

A limitation of the current study was that it was conducted using a small number of camera traps, and data concerning abundance and species density were not determined. The carnivore-to-noncarnivore ratio of 1:20 estimated by the number of camera trap pictures is likely biased for this reason. A prey preference biomass ratio for carnivores versus noncarnivores was estimated as 1:10 from similar locations within South Africa [88,89]. This prey preference ratio takes into account the morphological specifications of each carnivore species along with the social dynamics and hunting skills [88] and is therefore likely to be more accurate than a ratio of photographic detections.

The current study was performed as a baseline to determine disease transmission risks among carnivores within these two reserves. Specimen collection and testing were beyond the scope of this preliminary study. Another limitation was that some areas in both reserves were not accessible by road or foot and safety was an important consideration when selecting suitable camera trap locations. Despite the limitations, results suggest that camera traps would be useful as a validation tool or as an alternative census method to traditional call-up surveys. Follow-up studies are required to estimate disease prevalence, but management plans should consider annual vaccinations or vaccination prior to translocation to mitigate potential disease transmission risks.

## 5. Conclusions

Management is an important part of conservation, and a number of tools are available to manage protected areas. For example, camera traps should be considered as a validation tool or an alternative census method to the traditional carnivore call-up survey. Conservation management must also consider annual disease vaccinations or vaccination prior to carnivore translocation due to spatial overlap among species and possible disease transmission risks.

## Figures and Tables

**Table 1:** Multivariable logistic regression results for the prediction of carnivore species detection within Andover and Manyeleti nature reserves for the years 2015–2017.

Carnivore Group	Variable	Baseline (Comparison)	Coefficient	OR (95% CI)	*p*-Value
Any	Andover	Manyeleti	−0.62	0.54 (0.27–1.07)	0.077
	Spring	Other season	0.45	1.57 (1.14–2.16)	0.005
	Vehicles detected	No vehicles	0.08	1.08 (1.04–1.12)	<0.001
	Buffalo detected	No buffalo	0.74	2.10 (1.40–3.16)	<0.001
	Impala detected	No impala	0.76	2.13 (1.47–3.08)	<0.001
	Warthog detected	No warthog	0.62	1.86 (1.01–3.43)	0.046
Feline	Andover	Manyeleti	−0.06	0.94 (0.71–1.25)	0.679
	Hippo detected	No hippo	1.18	3.27 (1.34–7.98)	0.009
	Hare detected	No hare	1.60	4.96 (2.51–3.78)	<0.001
Canine	None significant				
Other	Andover	Manyeleti	0.29	1.34 (0.51–3.51)	0.555
	Altitude (meters)	N/A	−0.01	0.99 (0.98–1.0)	0.008
	Ant eater detected	No anteater	2.55	12.76 (1.02–159.15)	0.048
	Buffalo detected	No buffalo	0.83	2.30 (1.43- 3.71)	0.001
	Impala detected	No impala	0.77	2.17 (1.40–3.35)	0.001
	Warthog detected	No warthog	0.78	2.17 (1.08–4.38)	0.030
	Hare detected	No hare	1.60	4.93 (2.33–10.42)	<0.001

OR = odds ratio. CI = confidence interval. Any carnivore: lion, leopard, spotted hyena, cheetah, civet, serval, genet, wild dog. Feline carnivore: lion, leopard, cheetah, serval; canine carnivore: jackal, wild dog; other carnivore: spotted hyena, civet, genet.

**Table 2:** Spearman’s rho rank correlation between carnivore species and herbivore detection rates (detections per day) based on camera trap data collected from two protected areas in Mpumalanga Province, South Africa for the years 2015–2017.

	Lion	Leopard	Hyena	Jackal	Serval	Cheetah	Wild Dog	Civet	Waterbuck	Buffalo	Bosbok	Wildebeest	Impala	Warthog	Hare
Lion	1.000														
Leopard	0.063	1.000													
Hyena	0.497 **	0.248 *	1.000												
Jackal	0.114	0.206 *	0.218 *	1.000											
Serval	0.034	0.294 **	−0.010	0.180	1.000										
Cheetah	0.241 *	0.112	0.074	−0.049	−0.061	1.000									
Wild dog	0.114	0.151	0.065	0.316 **	0.061	0.214 *	1.000								
Civet	0.184	0.196	0.024	0.140	0.450 **	−0.040	0.166	1.000							
Waterbuck	0.097	0.294 **	0.145	0.102	0.136	0.102	0.014	0.183	1.000						
Buffalo	0.311 **	0.143	0.315 **	0.037	−0.019	0.165	0.093	0.081	0.314 **	1.000					
Bosbok	−0.078	0.406 **	0.101	−0.026	0.303 **	0.095	−0.005	0.284 **	0.352 **	0.025	1.000				
Wildebeest	0.385 **	0.089	0.253 **	0.202 **	0.052	0.043	−0.092	0.033	0.359 **	0.450 **	−0.034	1.000			
Impala	0.317 **	0.164	0.430 **	0.088	−0.123	0.271 **	−0.063	−0.016	0.374 **	0.439 **	0.042	0.473 **	1.000		
Warthog	0.282 **	0.220 *	0.293 **	0.017	0.097	0.279 **	0.014	0.211 *	0.245 *	0.404 **	0.222 *	0.372 **	0.442 **	1.000	
Hare	0.401 **	0.076	0.131	0.322 **	0.106	−0.065	0.051	0.263 *	0.298 **	0.284 **	−0.003	0.222 *	0.268 *	0.171	1.000

Significant correlations are highlighted with a background and ** indicates significance at the 0.01 level (two-tailed) and * significance at the 0.05 level.

## Data Availability

Data analyzed as part of this study are available at: https://figshare.com/s/ce58a61cef924c6d27a8 (accessed on 18 November 2020).

## Supplementary Materials

The following are available online at https://www.mdpi.com/article/10.3390/ani11092535/s1, Table S1: Summary of camera traps and census, Table S2: The significant univariate associations between any carnivore group (lion, leopard, serval, cheetah), canine (African wild dog, jackal), other (spotted hyena, civet, and genet) group detection and continuous predictor variables based on camera trap data collected within two protected areas, Mpumalanga Province, South Africa for the years 2015–2017., Table S3: The important univariate associations between lion, leopard, cheetah, serval, African wild dog, jackal, spotted hyena, civet, and genet detection and continuous predictor variables on camera trap data collected within two protected areas, Mpumalanga Province, South Africa for the years 2015–2017, Table S4: The important univariate associations between lion, leopard, serval, and cheetah detection and continuous predictor variables on camera trap data collected within two protected areas, Mpumalanga Province, South Africa, for the years 2015–2017, Table S5: The important univariate associations between spotted hyena, genet, and civet detection and continuous predictor variables on camera trap data collected within two protected areas, Mpumalanga Province, South Africa, for the years 2015–2017, Table S6: The important univariate associations between spotted hyena, detection, and continuous predictor variables on camera trap data collected within two protected areas, Mpumalanga Province, South Africa, for the years 2015–2017.
