# Supplementary material for: Carnivore Detection at the Domestic/Wildlife Interface within Mpumalanga Province, South Africa
Source: Animals (Basel). 2021 Aug 29;11(9):2535. doi: 10.3390/ani11092535 (PMC8466448; doi:10.3390/ani11092535)
Supplement: Supplementary file 1 [file animals-11-02535-s001.zip › animals-1319841-supplementary.pdf]

Supplemental Table S1. Summary of camera traps and census

Date	Call-up	Water	Lions	Leopard	Spotted Hyena	Wild Dog	Cheetah	Jackal	Lions	Leopard	Spotted Hyena	Wild Dog	Cheetah	Jackal
May-15	Call station 1	6 dams	n/a	n/a	n/a	n/a	n/a	n/a	0	1	0	0	0	0
	Call station 2	7 dams	n/a	n/a	n/a	n/a	n/a	n/a	0	0	1	0	0	0
	Call station 3	1 dam shared with call station 2	n/a	n/a	n/a	n/a	n/a	n/a	1	0	1	0	0	0
	Call station 4	6 dams	3	4	9	0	0	1	3	0	3	0	0	0
	Call station 5	7 dams share 4 with call station 4	0	0	0	0	0	0	0	1	2	0	0	0
	Call station 6	11 dams share 3 with call station 4 and 5	0	0	0	0	0	0	2	2	4	0	0	0
	Call station 7	5 dams	3	0	3	0	0	0	3	0	3	0	0	0
	Call station 8	3 dams share 2 with call station 7	8	1	9	n/a	n/a	n/a	0	0	4	0	0	0
Oct-15	Call station 1	6 dams	5	3	16	0	1	0	3	0	0	0	0	0
	Call station 2	7 dams	n/a	n/a	n/a	n/a	n/a	n/a	4	0	1	0	0	0
	Call station 3	1 dam shared with call station 2	n/a	n/a	n/a	n/a	n/a	n/a	0	0	3	0	0	0
	Call station 4	6 dams	0	0	0	0	0	0	1	0	0	0	0	0
	Call station 5	7 dams share 4 with call station 4	1	0	3	0	0	0	6	0	5	0	0	0
	Call station 6	11 dams share 3 with call station 4 and 5	8	1	9	0	0	0	0	0	0	0	2	0
	Call station 7	5 dams	n/a	n/a	n/a	n/a	n/a	n/a	0	1	2	0	0	0
	Call station 8	3 dams share 2 with call station 7	n/a	n/a	n/a	n/a	n/a	n/a	5	0	4	0	0	0

May-16	Call station 1	6 dams	n/a	n/a	n/a	n/a	n/a	n/a	0	2	0	0	0	0
	Call station 2	7 dams	n/a	n/a	n/a	n/a	n/a	n/a	0	0	1	0	0	0
	Call station 3	1 dam shared with call station 2	n/a	n/a	n/a	n/a	n/a	n/a	2	0	0	0	0	0
	Call station 4	6 dams	n/a	n/a	n/a	n/a	n/a	n/a	3	1	0	0	0	0
	Call station 5	7 dams share 4 with call station 4	n/a	n/a	n/a	n/a	n/a	n/a	1	0	0	0	0	0
	Call station 6	11 dams share 3 with call station 4 and 5	n/a	n/a	n/a	n/a	n/a	n/a	0	0	0	0	0	0
	Call station 7	5 dams	n/a	n/a	n/a	n/a	n/a	n/a	0	0	0	0	0	0
	Call station 8	3 dams share 2 with call station 7	n/a	n/a	n/a	n/a	n/a	n/a	0	0	1	3	0	0
Oct-16	Call station 1	6 dams	28	2	12	5	0	2	3	0	0	0	0	0
	Call station 2	7 dams	n/a	n/a	n/a	n/a	n/a	n/a	0	0	2	0	0	0
	Call station 3	1 dam shared with call station 2	n/a	n/a	n/a	n/a	n/a	n/a	0	0	0	0	0	0
	Call station 4	6 dams	0	0	5	0	0	0	3	1	0	0	0	0
	Call station 5	7 dams share 4 with call station 4	n/a	n/a	n/a	n/a	n/a	n/a	2	0	1	0	0	0
	Call station 6	11 dams share 3 with call station 4 and 5	9	0	9	0	0	0	2	1	0	0	0	0
	Call station 7	5 dams	n/a	n/a	n/a	n/a	n/a	n/a	0	0	2	0	0	0
	Call station 8	3 dams share 2 with call station 7	n/a	n/a	n/a	n/a	n/a	n/a	0	1	1	0	0	0
May-17	Call station 1	6 dams	n/a	n/a	n/a	n/a	n/a	n/a	0	0	0	0	0	0
	Call station 2	7 dams	0	1	0	0	0	0	3	0	1	0	0	0

Call station 3	1 dam shared with call station 2	n/a	n/a	n/a	n/a	n/a	n/a	4	0	0	0	0	0
Call station 4	6 dams	n/a	n/a	n/a	n/a	n/a	n/a	2	2	0	0	0	0
Call station 5	7 dams share 4 with call station 4	n/a	n/a	n/a	n/a	n/a	n/a	1	1	0	0	0	0
Call station 6	11 dams share 3 with call station 4 and 5	n/a	n/a	n/a	n/a	n/a	n/a	1	0	0	0	0	0
Call station 7	5 dams	n/a	n/a	n/a	n/a	n/a	n/a	0	0	1	0	0	1
Call station 8	3 dams share 2 with call station 7	n/a	n/a	n/a	n/a	n/a	n/a	1	0	1	0	0	0
Totals		65	12	75	5	1	3	56	14	44	3	2	1

camera traps within a 5 km range were used on the day of the call-up for the summary.

Table S2. The significant univariate associations between any carnivore group (lion, leopard, serval, cheetah), canine (African wild dog, jackal), other (spotted hyena, civet, and genet) group detection and continuous predictor variables based on camera trap data collected within two protected areas, Mpumalanga Province, South Africa for the years 2015–2017.

Group	Variable	Compared too	Coefficient	OR (95% CI)	*p*-value
Any	Spring	Summer	0.39	1.48(0.97–2.25)	0.067
	Winter	Summer	-0.17	0.84 (0.53–1.34)	0.471
	Autumn	Summer	0.19	1.21 (0.78–1.86)	0.410
Feline	First quarter	Full moon	0.32	1.38 (0.85–2.23)	0.199
	New moon	Full moon	0.08	0.93 (0.55–1.57)	0.782
	Third moon	Full moon	-0.01	0.99 (0.59–1.66)	0.967
	Hippo detected	No hippo	1.77	5.89 (3.27–15.26)	<0.001
	Giraffe detected	No Giraffe	1.09	2.96 (1.40–6.27)	<0.005
	Waterbuck detected	No waterbuck	1.05	2.87 (1.07–7.69)	0.036
	Buffalo detected	No buffalo	0.61	1.83 (0.98–3.43)	0.057
	Bosbok detected	No bosbok	1.00	2.72 (0.79–9.34)	0.113

	Njala detected	No njala	2.24	9.42 (1.92–46.24)	0.006
	Impala detected	No impala	0.53	1.70 (0.94–3.07)	0.082
	Elephant detected	No elephant	0.41	1.51 (0.90–2.54)	0.122
	Warthog detected	No warthog	0.72	2.03 (0.84–4.92)	0.118
	Hare detected	No hare	1.70	5.49 (2.82–10.68)	<0.001
Other	Spring	Summer	0.27	1.32 (0.77–2.25)	0.316
	Winter	Summer	-0.01	0.99 (0.56–1.79)	0.980
	Autumn	Summer	0.61	1.85 (1.08–3.16)	0.025
	Ant eater detected	No Anteater	2.73	15.37 (1.29–138.69)	0.031
	Waterbuck detected	No Waterbuck	1.04	2.83 (1.12–7.13)	0.028
	Giraffe detected	No giraffe	0.62	1.85 (0.95–3.60)	0.070
	Elephant detected	No elephant	0.41	1.51 (0.99–2.30)	0.058
	Buffalo detected	No buffalo	0.99	2.70 (1.70–4.29)	<0.001
	Wildebeest detected	No wildebeest	0.87	2.39 (1.20–4.73)	0.013
	Rhino detected		0.67	1.95 (1.04–3.65)	0.037
	Impala detected	No impala	0.93	2.53 (1.64–3.89)	<0.001
	Grey duiker detected	No Grey duiker	0.70	2.02 (1.10–3.72)	0.024
	Goat outside detected	No goat outside	2.38	10.75 (0.81–142.28)	0.072
	Warthog detected	No warthog	0.97	2.64 (1.35–5.18)	0.009
	Hare detected	No hare	1.71	5.53 (2.67–11.48)	<0.001
Specie: Spotted Hyena	Elephant detected	No elephant	0.39	1.48 (0.96–2.29)	0.078
	Wildebeest detected	Wildebeest	0.88	2.41 (1.21–4.80)	0.012
	Grey Duiker detected	Grey Duiker	0.46	1.59 (0.81–3.12)	0.177
	Impala detected	Impala	0.96	2.61 (1.97–4.02)	<0.001
	Warthog detected	Warthog	1.06	2.88 (1.47–5.68)	0.002
	Hare detected	Hare	1.37	3.94 (0.57–2.18)	0.001
	Buffalo detected	Buffalo	0.94	2.55 (1.58–4.12)	<0.001

Any Carnivores: lion, leopard, cheetah, spotted hyena, civet, serval, wild dog, genet. Feline carnivores: lion, leopard, cheetah, serval. Other carnivores: spotted hyena, civet, genet.

Table S3. The important univariate associations between lion, leopard, cheetah, serval, African wild dog, jackal, spotted hyena, civet, and genet detection and continuous predictor variables on camera trap data collected within two protected areas, Mpumalanga Province, South Africa for the years 2015–2017.

Variable	Coefficient	OR (95% CI)	*p*-Value
Longitude	2.25	9.46 (0.60–149.23)	0.110
Altitude(meters)	-0.01	0.99 (0.99–1.00)	0.003
Open areas	0.66	1.94 (0.88–4.28)	0.103
Dam/rivers	-0.57	0.57 (0.24–1.34)	0.196
Rain(mm)	-0.04	0.96 (0.91–1.00)	0.057
Rain previous day	-0.03	0.97 (0.93–1.01)	0.185
Vehicles detected	0.08	1.08 (1.04–1.13)	<0.001
Giraffe detected	0.69	1.99 (1.16–3.45)	0.013
Buffalo detected	0.77	2.16 (1.45–3.23)	<0.001
Wildebeest detected	0.60	1.81 (1.00–3.30)	0.051
Impala detected	0.80	3.25 (1.56–3.24)	<0.001.
Warthog detected	0.77	2.17 (1.20–3.93)	0.011

Any carnivore: lion, leopard, cheetah, spotted hyena, genet, civet, wild dog, serval.

Table S4. The important univariate associations between lion, leopard, serval, and cheetah detection and continuous predictor variables on camera trap data collected within two protected areas, Mpumalanga Province, South Africa, for the years 2015–2017.

**Variable**	**Coefficient**	**OR (95% CI)**	***p*-Value**
Minimum temperature(°C)	-0.03	0.97 (0.92–1.02)	0.196
Rain(mm)	-0.06	0.94 (0.86–1.03)	0.164

Feline carnivores: lion, leopard, serval, cheetah

Table S5. The important univariate associations between spotted hyena, genet, and civet detection and continuous predictor variables on camera trap data collected within two protected areas, Mpumalanga Province, South Africa, for the years 2015–2017.

**Variable**	**Coefficient**	**OR (95% CI)**	***p*-Value**
Longitude	2.74	15.47 (1.16–205.56)	0.038
Altitude(meters)	-0.01	0.99 (0.99–1.00)	<0.001

Rain(mm)	-0.07	0.94 (0.89–1.01)	0.081
Summer	0.53	1.69 (1.12–2.55)	0.012
Vehicles detected	0.03	1.03 (0.99–1.07)	0.117

Other carnivores: spotted hyena, genet, civet.

Table S6. The important univariate associations between spotted hyena, detection, and continuous predictor variables on camera trap data collected within two protected areas, Mpumalanga Province, South Africa, for the years 2015–2017.

Variable	Coefficient	OR (95% CI)	*p*-Value
Longitude(meters)	3.11	22.39 (1.55–324.08)	0.023
Altitude(meters)	-0.01	0.99 (0.99–1.00)	<0.001
Rain(mm)	-0.06	0.95 (0.89–1.01)	0.090
Vehicles detected	0.03	0.13 (-0.01–0.06)	0.130
